# B-Cell Lymphoma in the Tricuspid Valve

**DOI:** 10.7759/cureus.930

**Published:** 2016-12-16

**Authors:** Ali C Agha, Joseph Limback, Raul Loya, Ashley Ramirez, Michael Valente, Jeremy Burt

**Affiliations:** 1 College of Medicine, University of Central Florida; 2 Diagnostic Radiology, Florida Hospital-Orlando

**Keywords:** diffuse large b-cell lymphoma (dlbcl), coronary ct angiography, tricuspid valve, coronary arteries, cardiac lymphoma

## Abstract

Lymphoma can involve any organ or tissue that contains lymphoid tissue and the heart is no exception. A few prior case reports have described lymphoma encasing a coronary artery or involving one or more cardiac valves. We present a rare case of diffuse large B-cell lymphoma (DLBCL) involving the tricuspid valve and right coronary artery diagnosed on coronary CT angiography. The clinical and imaging characteristics of cardiac lymphoma are discussed.

## Introduction

Lymphoma can involve any organ or tissue that contains lymphoid tissue and the heart is no exception. Occasionally, lymphoma will infiltrate the pericardium or myocardium. In rare circumstances, lymphoma encases a coronary artery or involves one or more cardiac valves. We present a rare case of a woman with diffuse large B-cell lymphoma (DLBCL) involving the tricuspid valve and right coronary artery.

## Case presentation

A 78-year-old female went to her primary care physician (PCP) complaining of progressively worsening shortness of breath. Her PCP referred her to the nearest emergency room to evaluate for possible pulmonary embolism. There was also concern that the patient may have chronic obstructive pulmonary disease (COPD) or asthma. She was given IV methylprednisolone and albuterol with an improvement of symptoms.

While in the emergency room, a CT angiogram of the chest was negative for pulmonary embolism but did reveal a right heart mass. A subsequent echocardiogram confirmed a mass-like structure centered in the tricuspid valve consistent with either a tumor or thrombus. She was started on IV heparin and transferred to our hospital.

A detailed history and physical examination were obtained at our institution. The patient had a past medical history, including Type 2 diabetes, hypertension, restless leg syndrome, and cerebrovascular accident (CVA) in 2014, that did not produce any neurologic deficit.

After the CVA, severe stenosis was discovered in her right carotid artery during imaging performed at an outside hospital and she underwent right carotid endarterectomy. During the surgery, a mass was visualized surrounding her right carotid artery, which was biopsied and confirmed to be diffuse large B-cell lymphoma (DLBCL). The patient underwent three cycles of chemotherapy and 17 treatments with radiation therapy resulting in complete remission.

There was also a brief smoking history, but she reported quitting over 35 years ago. The physical exam was unremarkable except for a presystolic murmur at the left sternal edge.

The only laboratory value abnormalities included a low hemoglobin (11.1) and normal mean cell volume (91.2), consistent with a normocytic anemia. Flow cytometry of peripheral blood detected no monoclonal B-cells, immunophenotypically abnormal T cells, or immature monocytes or melanocytes. Peripheral smear revealed a normocytic anemia consistent with the laboratory abnormalities.

**Figure 1:**
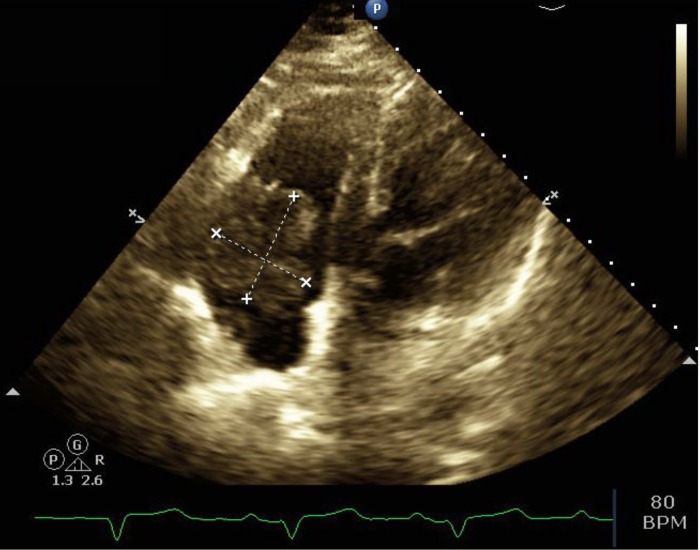
Four chamber echocardiogram demonstrating a large, mobile echodensity measuring 4.39cm x 4.12cm in the tricuspid valve and anterior atrioventricular wall, extending into the right atrium and ventricle.

An echocardiogram and coronary CT angiogram were performed at our institution to better define the cardiac mass. The echocardiogram demonstrated normal left ventricular chamber size, wall motion, and contractility with an ejection fraction of 65%. The left atrial chamber size was normal. A large, mobile, hypoechoic mass was identified in the tricuspid valve measuring 4.39 cm x 4.12 cm (Figure 1). Tricuspid regurgitation and mild dilatation of the right atrium were also visualized.

**Figure 2:**
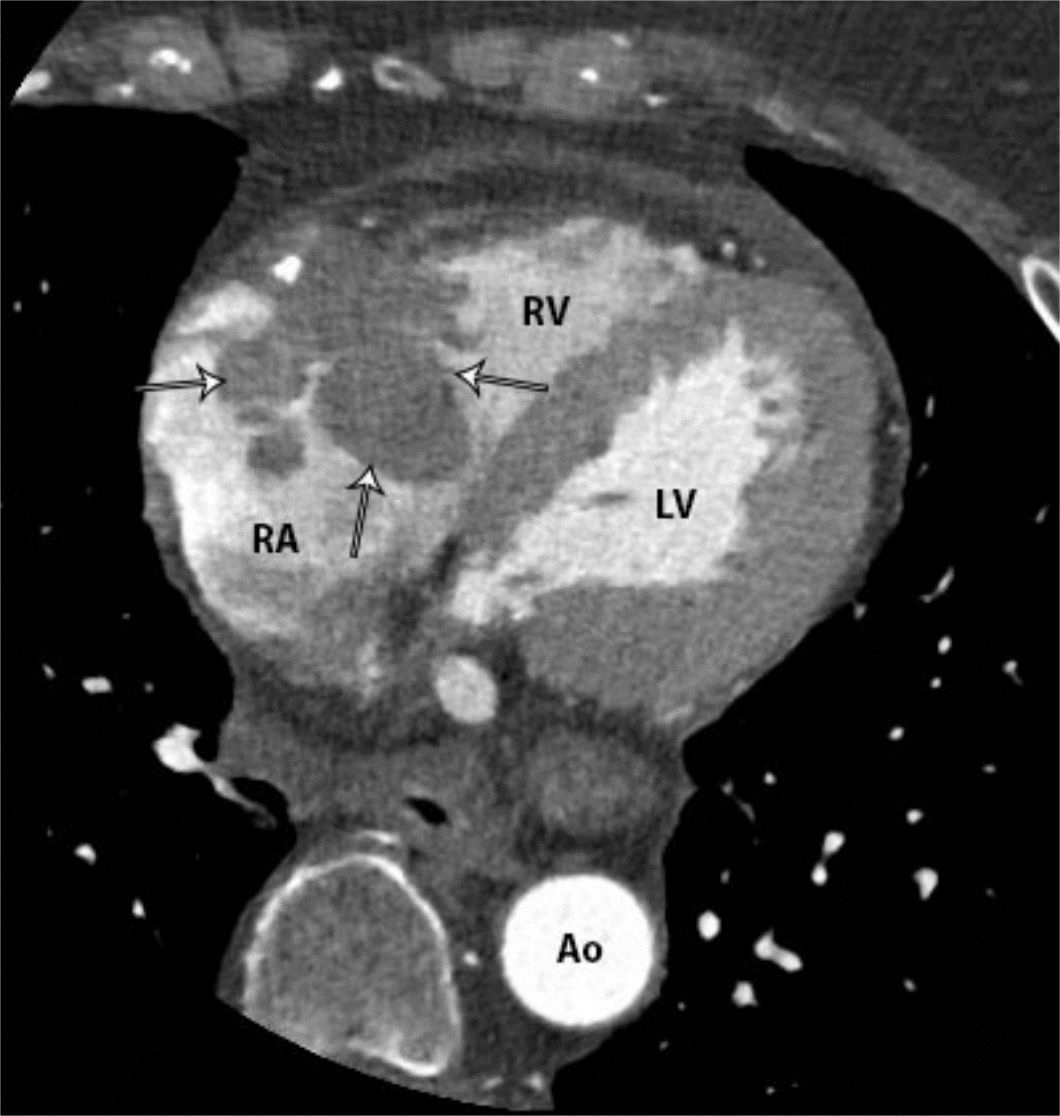
Axial coronary CT angiogram with contrast demonstrating a tricuspid mass (white arrows) extending into the right atrium and ventricle at the level of the AV canal, with narrowing of the right atrioventricular orifice and mild dilation of the right atrium. RA=right atrium; RV=right ventricle; LV=left ventricle; Ao=descending aorta.

**Figure 3:**
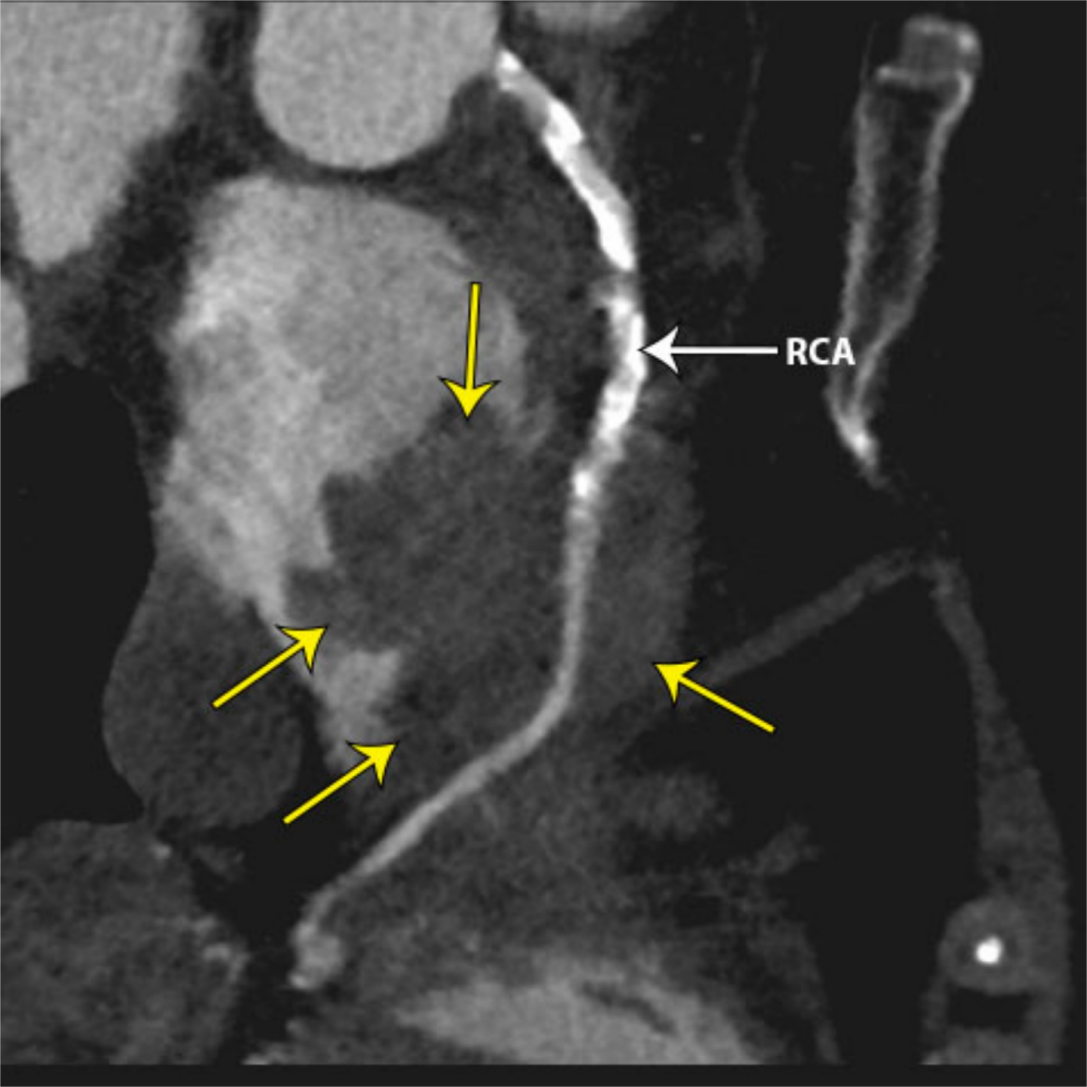
Curviplanar reformatted image of a coronary CT angiogram demonstrating a large, enhancing mass (yellow arrows) encasing most of the right coronary artery (RCA).

**Figure 4:**
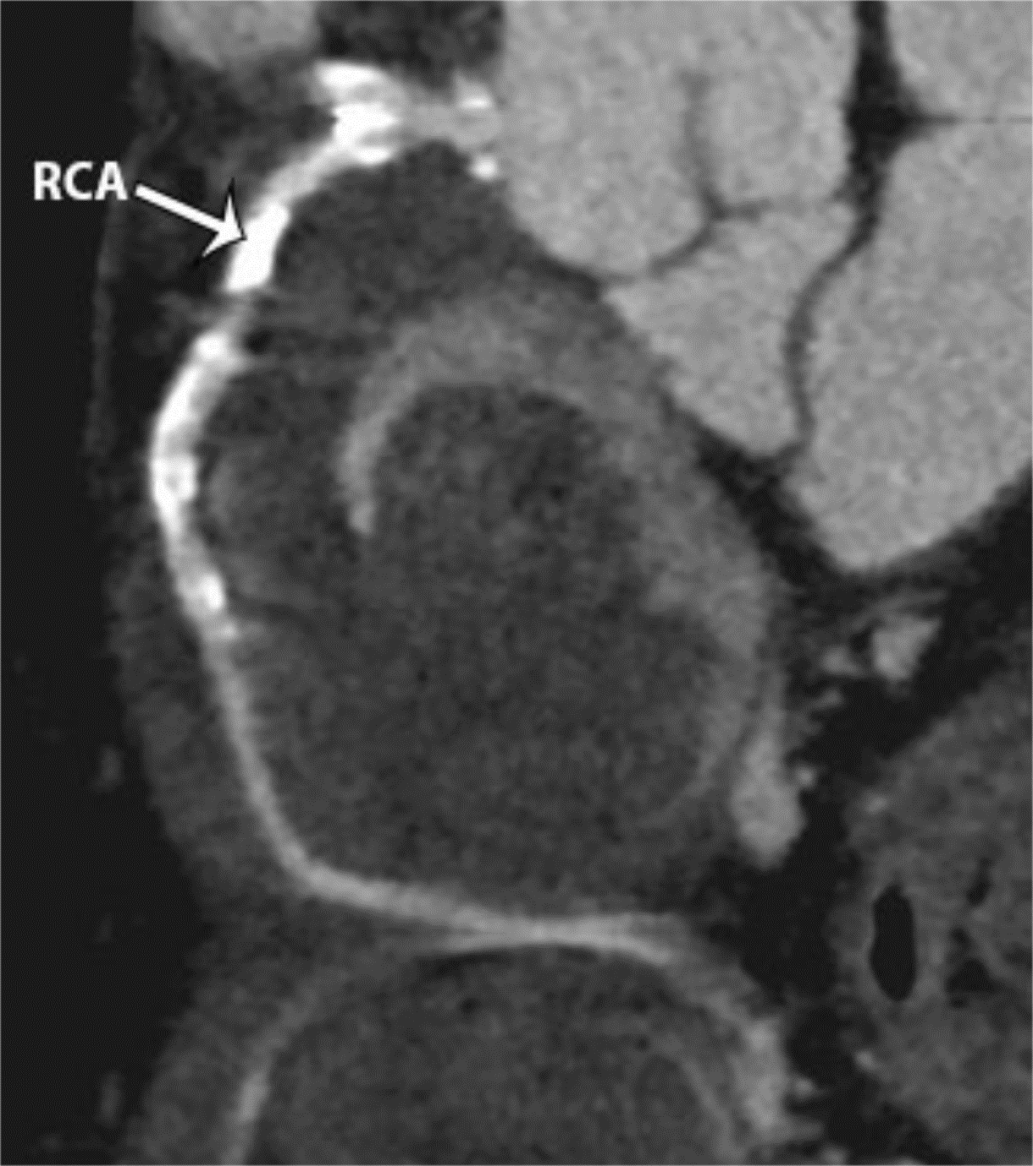
Another curviplanar reformatted image of lymphoma surrounding the right coronary artery (RCA).

**Figure 5:**
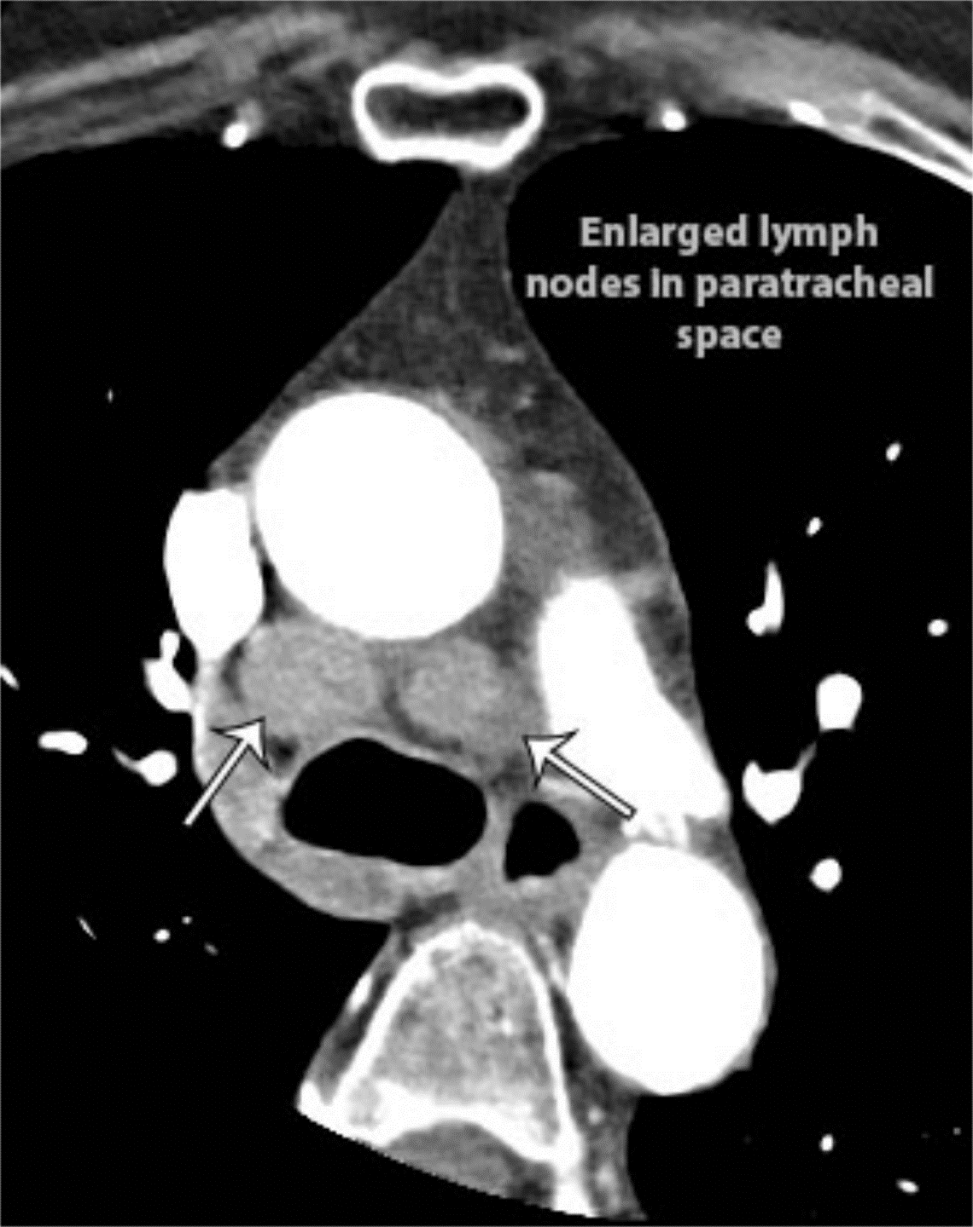
Axial coronary CT angiogram with contrast.

Coronary CT angiography demonstrated a large enhancing mass centered in the anterior AV groove and tricuspid valve, surrounding most of the right coronary artery, and extending into the right atrium and right ventricle at the level of the atrioventricular (AV) canal (Figure 2). There was narrowing of the right atrioventricular orifice and mild dilation of the right atrium. The right coronary artery was completely encased with tumor but had no associated stenosis (Figure 3). Multifocal calcified atherosclerotic plaque was also identified in the coronary arteries (Figure 4). Visibly enlarged mediastinal and right hilar lymph nodes were also evident (Figure 5).

Given the history of DLBCL in the neck, the constellation of imaging findings was compatible with a diagnosis of recurrent lymphoma in the heart.

Although tissue diagnosis was recommended to confirm the cell type and perform flow cytometry, the patient refused any further invasive diagnostic procedures or aggressive treatment and opted for palliative care. The patient expired one week later. 

## Discussion

Diffuse large B-cell lymphoma (DLBCL) is the most common type of non-Hodgkin lymphoma (NHL) affecting adults, comprising approximately 25% of cases. The histologic appearance distinguishes this type of NHL from others, demonstrating transformed B-cells with a basophilic cytoplasm and prominent nucleoli. Tumor cells can often be detected by flow cytometry demonstrating increased expression of B-cell antigens (CD19, CD20, CD22, CD79a) [1].

Common symptoms of DLBCL include a rapidly enlarging mass and lymph node enlargement in the neck or abdomen. Constitutional symptoms known as “B” symptoms, including fever, weight loss, and night sweats, occur in many patients. However, many cases of cardiac lymphoma go undetected and are not identified until death [2]. In an autopsy study of patients who were diagnosed with malignant lymphoma during their lifetimes, 16% demonstrated cardiac involvement despite the fact that cardiac lymphoma rarely presents clinically [3].

Metastatic disease to the heart is more common than primary cardiac neoplasia [4]. Common primary tumors metastasizing to the heart are pleural mesothelioma, melanoma, lung adenocarcinoma, and undifferentiated carcinoma [5]. Lymphoma and leukemia are much less common. Infiltration of the tricuspid valve by lymphoma is unusual with only five prior cases reported. No prior cases of lymphomatous involvement of both the tricuspid valve and right coronary artery have been reported.

Lymphoma can metastasize to the heart by any of the following mechanisms: direct extension, hematogenous spread, lymphatic spread, and intracavitary diffusion (by means of the inferior vena cava or pulmonary vein) [4].

Although cardiac lymphoma often remains asymptomatic, symptoms may arise due to valvular involvement or decreased cardiac function after infiltration of the heart or its surrounding pericardium. Non-Hodgkin’s lymphoma (NHL) tends to affect the myocardium, where neoplastic infiltration can replace myocardial tissue. In contrast, Hodgkin’s lymphoma often affects the pericardium, sometimes causing malignant pericardial effusion or tamponade [4, 6]. Lymphoma may also affect the conduction system of the heart leading to arrhythmias.

DLBCL is typically diagnosed by excisional tissue biopsy of a lymph node. However, one unique characteristic of a proliferating lymphoma on imagining is its tendency to surround structures, such as blood vessels, but not directly invade them [7]; this was the basis for the diagnosis of recurrent lymphoma in our patient.

Chemotherapy regimens, such as R-CHOP (rituximab, cyclophosphamide, doxorubicin hydrochloride, Oncovin®, and prednisone), have been shown to be beneficial in some cases of cardiac lymphoma [8]. CT/FDG-PET may be employed to assess the response of cardiac lymphoma to chemotherapy [9-10].  

This research study was approved by the Institutional Review Board (IRB) of Florida Hospital and informed consent was waived.

## Conclusions

Cardiac involvement by lymphoma is unusual. We report a rare case of diffuse large B-cell lymphoma involving the tricuspid valve, anterior atrioventricular wall, and right coronary artery diagnosed on coronary CT angiography. The patient elected for palliative management and died one week after imaging diagnosis.
